# A Review of the Regulatory Challenges of Personalized Medicine

**DOI:** 10.7759/cureus.67891

**Published:** 2024-08-27

**Authors:** Dharani S, Kamaraj R

**Affiliations:** 1 Pharmacy, SRM College of Pharmacy, SRM Institute of Science and Technology (SRMIST), Chennai, IND

**Keywords:** molecular diagnostics, genomic research, targeted therapies, precision medicine, pharmacogenomics

## Abstract

Personalized medicine integrates genomics with clinical and familial histories, revolutionizing healthcare by tailoring treatments to individual patient characteristics. At its core, pharmacogenomics enables the customization of medication prescriptions based on genetic profiles, enhancing drug efficacy and safety. This precision medicine approach addresses disease diagnosis, prevention, and treatment, offering targeted therapies for conditions like autoimmune disorders, rheumatoid arthritis, and neoplastic conditions. Examples of pharmacogenomics and personalized medicine include treatment for certain conditions like blood clotting disorders (warfarin (blood thinner), genetic variability, acute lymphoblastic leukemia (ALL), and thiopurine methyltransferase (TPMT) testing) in leukemia treatment.

Historically, personalized medicine has evolved from Hippocrates' humoral theories to modern molecular diagnostics. The shift from cellular to molecular-level investigations has led to the current post-genomic era, emphasizing the four chemical components of DNA in understanding and treating disorders. This evolution enhances our ability to predict disease susceptibility, treatment response, and potential adverse reactions, demanding advancements in privacy laws, payment systems, regulatory standards, and education.

Personalized healthcare optimizes treatment by considering genetic, environmental, and lifestyle factors, reducing adverse reactions, and improving patient satisfaction. It drives genomic and biotechnological research, fostering the development of targeted therapies and diagnostic tools, and streamlining drug development.

Applications in lung cancer, renal carcinoma, and rheumatoid arthritis (RA) illustrate the efficacy of personalized medicine. Targeted therapies, such as tyrosine kinase inhibitors (TKIs) and monoclonal antibodies (mAbs), show significant success in lung cancer. Biomarkers guide renal carcinoma treatments, while gene expression profiles predict rheumatoid arthritis outcomes with tumor necrosis factor-alpha* (*TNF-α) blockers.

The US FDA's increasing approvals of personalized treatments underscore its commitment to precision medicine. However, regulatory challenges persist, necessitating clear processes and policies. Ethical considerations, including equitable access and privacy, are critical for responsible implementation. The FDA's increasing approvals of personalized treatments highlight its commitment to advancing precision medicine, yet regulatory challenges remain, requiring the development of clear processes and policies to manage innovation safely. While these hurdles are being addressed through evolving guidelines and collaborative efforts, ethical considerations, including equitable access and privacy, are critical for responsible implementation. Early examples, such as warfarin and imatinib, demonstrate the role of genetic information in guiding treatment, illustrating the potential of personalized medicine to optimize healthcare outcomes.

## Introduction and background

A personalized approach to healthcare involves integrating advancements in genomics with clinical and family histories to effectively tailor treatments for patients. A fundamental aspect of healthcare is the application of pharmacogenomics principles in clinical settings. Pharmacogenomics is increasingly recognized for its potential to customize medication prescriptions for specific subgroups or even individuals based on their unique characteristics, ultimately enhancing drug efficacy and safety [1].

Personalized medicine, synonymous with precision medicine, focuses on tailoring care according to each patient's traits. This approach relies on utilizing genomic information to address concerns related to disease prevention, treatment, and diagnosis. Grounded in pharmacogenomics and genomics, personalized medicine offers targeted therapies that benefit a wide range of conditions, including brain tumors, rheumatoid arthritis (RA), prostate cancer, autoimmune disorders, and lung cancer, among others [2].

The history of personalized medicine is less concerned with the initial development of the concept of "personalization," first introduced by Hippocrates (circa 460 Before the Christian Era (BCE)-circa 370 BCE) approximately 2,400 years ago, and more focused on the evolution and increasing precision of diagnosis and treatment. As medical advancements have progressed, the methodologies and instruments for elucidating and identifying illnesses have transitioned from conceptual frameworks to empirical observations. This evolution has shifted the focus from cellular-level investigations to molecular-level details and, more recently, to understanding how molecular processes interact with broader aspects such as cognition and behavior.

The objective of personalizing healthcare has remained consistent over time, with each era introducing new tools for customizing treatment for individuals. In ancient times, personalized medicine was based on the concept of the four humors-blood, phlegm, yellow bile, and black bile-where treatments were tailored to balance these humors according to each individual’s unique constitution and symptoms. Today, we rely on the four chemical components of DNA to understand and treat disorders through brain imaging and neurotransmitter actions. As we trace the evolution of medicine from antiquity to the present, we witness a significant transition into what can be termed the post-genomic era. The post-genomic era focuses on using detailed genetic and molecular information to personalize healthcare. It integrates advanced technologies to better understand individual health risks and tailor treatments more precisely. With molecular diagnostics and functional markers, we can now better predict an individual's risk of disease, how they will respond to treatments, and possible side effects well before they occur.

The advent of medical prediction will necessitate concurrent advancements in various aspects of healthcare and society, including privacy laws, payment systems, regulatory standards, education for healthcare providers and patients, and ethical frameworks. Exploring the journey that has shaped medicine as it stands today offers insights into what lies ahead in our near future [3].

Tailoring medical treatments to individual profiles, a concept rooted in ancient practices of balancing the four humors has evolved significantly in modern medicine. Today, personalized medicine uses advanced genetic and molecular information to enhance treatment effectiveness and minimize side effects, reflecting a transition from early, generalized approaches to precise, individualized care. This approach allows for the prevention of diseases and streamlines healthcare by eliminating the need for trial and error in selecting therapies [4]. The future of personalized medicine will focus on advancing genomics and artificial intelligence (AI), with adaptive policies ensuring innovation, safety, and fairness. Collaboration and ethical considerations will be crucial for its successful integration into healthcare.

## Review

Importance

Personalized healthcare enhances treatment effectiveness through the customization of therapies based on genetic, environmental, and lifestyle factors. By selecting medications that align with a patient's biological characteristics, personalized medicine mitigates the risk of side effects. For example, trastuzumab is used for HER2-positive breast cancer to reduce side effects, while genetic tests guide statin use to manage cholesterol and adjust warfarin doses to prevent bleeding risks, offering safer treatment options. This approach facilitates proactive and preventive healthcare by identifying at-risk individuals and implementing timely interventions. Personalized medicine is cost-effective despite its initial expenses. It prevents unnecessary effects and minimizes ineffective therapy, which maximizes the use of resources for maintaining health. It fosters patient-centered care through individualized treatment plans, enhancing patient satisfaction and adherence [5].

Moreover, personalized medicine drives advances in genomic and biotechnological research and technology, facilitating the development of targeted therapies and diagnostic tools. Personalized medicine streamlines drug development processes, lowering trial failure rates and expediting treatment availability. Ultimately, personalized medicine helps classify diseases by their molecular traits. This leads to accurate diagnoses and customized treatments that improve patient outcomes.

Applications

Personalized Medicine for Lung Cancer

The therapy landscape for lung cancer includes various treatment options, such as chemotherapy, immunotherapy, and targeted therapies. Chemotherapy uses drugs to kill cancer cells or stop their growth, while immunotherapy harnesses the body's immune system to fight cancer. Targeted therapies, such as monoclonal antibodies (mAbs) and tyrosine kinase inhibitors (TKIs) that focus on specific proteins like the epidermal growth factor receptor (EGFR), are also widely used and have shown a favorable response rate exceeding 70%. These therapies have demonstrated a favorable response rate exceeding 70%. However, ongoing research suggests that targeted therapies can occasionally intensify the condition compared to conventional drugs, and they are generally associated with higher costs. For example, some patients treated with targeted therapies like osimertinib (a third-generation EGFR inhibitor) for non-small cell lung cancer (NSCLC) have reported disease progression or resistance over time, necessitating alternative treatments. Additionally, the high cost of targeted therapies, such as the mAb nivolumab used in immunotherapy, poses financial challenges for both patients and healthcare systems. Non-small cell lung cancer constitutes about 85% of all lung cancer cases and is characterized by abnormal cellular growth. Personalized medicine in cancer treatment involves employing positron emission tomography (PET) with radiolabeled drugs to evaluate metabolite concentrations and pharmacokinetics (PKs) through blood and urine analyses. Positron emission tomography imaging utilizes gamma rays to generate targeted images by administering radiolabeled drugs, facilitating the development of tailored treatments. Pharmacokinetics studies of these drugs aid in optimizing medication formulations and predicting the binding of radiolabeled drugs to tumors [6].

Immuno-PET, employing radioisotope-labeled mAbs such as 89Zr, is commonly utilized in antigen therapy. Due to the slow PKs of mAbs, dynamic scanning is impractical, necessitating static imaging relying on tumor-to-blood ratios and standardized uptake values (SUVs) relative to reference tissues. Upon mAb binding, target cells internalize the antibodies. The internalization of mAbs by target cells is crucial because it allows the antibodies to deliver therapeutic agents directly into the cells. This process enhances the effectiveness of treatments by ensuring that the therapeutic agents are precisely delivered to the intended targets, improving the overall efficacy of the therapy.

Radioactive particles like iodine-124 and bromine-76 undergo decay and cellular elimination. The TKI-PET method is employed to visualize affected cells, focusing specifically on EGFR.

The TKIs, small molecules that inhibit kinases crucial for cell proliferation, function by competing with adenosine triphosphate (ATP) to bind to their target tyrosine kinase. For example, erlotinib (which blocks ATP binding in EGFR and is also used for EGFR overexpression by blocking the EGFR kinase) targets the kinase domain of EGFR by displacing AT [7]. Erlotinib (Tarceva) has improved outcomes for patients with advanced NSCLC. It helps reduce tumor size and extends survival, especially in those with specific genetic mutations. Compared to chemotherapy, it often has fewer side effects and a better quality of life for patients, more examples, such as imatinib (Gleevec), which is used in treating chronic myeloid leukemia (CML), targets the BCR-ABL tyrosine kinase, which is active in CML cells. Sunitinib (Sutent) targets multiple kinases, including those involved in tumor growth and angiogenesis, used for renal cell carcinoma (RCC) and gastrointestinal stromal tumors. Gefitinib (Iressa), another EGFR inhibitor used for NSCLC, blocks the tyrosine kinase activity involved in cancer cell proliferation. The TKIs are typically radiolabeled with 18F or 11C due to their short half-lives, facilitating alignment with biological processes. This short duration helps ensure that imaging aligns closely with the biological processes of the drug, improving the timing and effectiveness of monitoring and treatment planning. Upon injection, radiolabeled TKIs rapidly bind to target cells and are often eliminated via renal pathways.

Dynamic scanning is frequently employed for PK modeling and quantitative tracking of uptake in vivo. The use of short-lived nucleotides allows for metabolism and enables patients to undergo repeated therapy shortly after initial treatment [8]. Personalized medicines that are sold are represented in Table 1.

**Table 1:** Marketed personalized medicines

Trade name	Class of drugs	Generic name	Target site
Tarceva	Tyrosine kinase inhibitor	Erlotinib	Epidermal growth factor receptor
Iressa	Tyrosine kinase inhibitor	Gefitinib	Epidermal growth factor receptor
Tagrisso	Tyrosine kinase inhibitor	Osimertinib	Epidermal growth factor receptor
Portrazza	Monoclonal antibody	Necitumumab	Epidermal growth factor receptor
Avastin	Monoclonal antibody	Bevacizumab	Vascular endothelial growth factor receptor
Cyramza	Monoclonal antibody	Ramucirumab	Vascular endothelial growth factor receptor
Keytruda	Monoclonal antibody	Pembrolizumab	Progressive disease

Personalized Medicine for Renal Carcinoma 

Europe is currently grappling with a substantial health challenge in renal carcinoma, which manifests in two main types: nephroblastoma and RCC. Annually, thousands of individuals receive diagnoses of tumors, with nephroblastoma, also known as Wilms tumor (WT), being the predominant type. Common symptoms include pain and swelling, particularly affecting children.

The Children's Oncology Group (COG) has identified a biomarker associated with nephroblastoma. Patients in stages I and II have been treated with a combination of doxorubicin (DOX), vincristine (VCR), and actinomycin D (ACT D). Regrettably, these treatment regimens have not yielded significant improvements in outcomes [9].

During clinical investigations, researchers noted that concentrating on the insulin-like growth factor (IGF) pathway with IMC A12, a mAb, led to a cessation in cell division and a reduction in tumor size. These findings showed promising therapeutic effects that could potentially benefit patients with rhabdomyosarcoma. Weigel et al. focused their research on evaluating the efficacy of cixutumumab, administered at a dose of 9 mg/kg, which was well-tolerated by children, suggesting its potential as an IGF-inhibiting agent [10].

Approximately 50%-70% of cases of renal cell carcinoma (ccRCC) involve Von Hippel Lindau (VHL) as a biomarker, along with markers like anhydrase and vascular endothelial growth factor (VEGF). Newly discovered personalized medicines such as bevacizumab, axitinib, sorafenib, and temsirolimus are tailored for treating these conditions. Despite these advances, some individuals may develop resistance to these medications, with sunitinib remaining a primary first-line treatment. To address resistance issues and enhance the efficacy of RCC therapy, combining drugs with complementary agents is recommended.

Xu et al. treated a patient with pazopanib and observed a correlation between a specific genetic variation (single nucleotide polymorphism (SNP)) and improved survival without progression, as well as an enhanced response compared to previous treatments [11].

Personalized Medicine for RA

The primary objective of the study conducted by Koczan et al. was to identify biomarkers that could predict therapeutic outcomes in RA patients treated with the tumor necrosis factor-alpha (TNF-α) blocker, etanercept. Nineteen RA patients were selected, including seven non-responders and 12 responders. Changes in preexisting gene expression were monitored using the Affymetrix microarray, and results were contrasted with after-treatment information. After 72 hours of treatment, gene expression changes were observed, correlating with an improved Disease Activity Score (DAS28 >1.2). It was noted that some patients developed interferon (IFN), a protein involved in the immune response. The presence of IFN was associated with a poorer response to the TNF-α blocker, etanercept. This suggests that IFN production might negatively impact the effectiveness of the treatment in those patients. The study concluded that using gene expression patterns to predict RA response early in TNF-α blocker treatment is promising. This approach could lead to more personalized medication strategies based on individual genetic profiles. However, potential limitations include the small sample size, which may affect the generalizability of the findings and the variability in how gene expression changes are interpreted across different patients. Additionally, the presence of interferon and its impact on treatment response highlights the need for further research to understand its role and develop strategies to mitigate its negative effects. [12].

Laquerre et al. examined the effects of combining infliximab with methotrexate and tracked results after three months. Thirteen patients were chosen for gene profiling using peripheral blood mononuclear cells. Among these patients, seven were non-responders and six were responders. Baseline RNAs were hybridized, and mRNA transcription was analyzed. Differences in transcription levels were compared with treatment responses, revealing downregulation in non-responders. A total of 279 genes exhibited differential expression between responders and non-responders [13].

FDA's Approval of Personalised Medicine

With the development of modern medicine, there has been a shift in the focus towards using molecular information to customize therapies for specific individuals. This approach has resulted in a significant increase in approvals for precision medicine by the US FDA. In 2023, personalized medicines made up more than one-third of all drug approvals, maintaining the same proportion for the fourth consecutive year. These approvals included 26 personalized treatments, comprising 20 molecular entities and six newly approved gene- and cell-based therapies. 

The rise in precision medicine approvals over the years underscores the FDA's commitment to supporting therapies that use molecular data to customize patient treatments. The CDER endorsed a total of 45 drugs in 2015, with 13 classified as precision medicines by the Personalized Medicine Coalition (PMC), representing over a quarter of approvals. Of the 41 medications authorized by the FDA in 2014, nine (or 20% of the total) were approved as precision medicine products. As to the PMC, personalized medicine pertains to medicines that have biomarkers on their labels that are specifically designed to support treatment methods and decision-making for individual patients. The status of approved personalized drugs is represented in Table 2 [2]. 

**Table 2:** Status of approved personalized drugs

Drug name	Disease	Approval year
Crizotinib	Non-small cell lung cancer	2013
Erlotinib	Non-small cell lung cancer	2013
Lynparza	Ovarian cancer	2014
Vimizim	Mucopolysaccharidosis	2014
Cyrazma	Non-small cell lung cancer	2014
Zykadia	Non-small cell lung cancer	2014
Beleodaq	T-cell lymphoma	2014
Cerdelga	Gaucher disease type I	2014
Harvoni	Hepatitis C	2014
Viekera	Hepatitis C	2014
Blincyto	Acute lymphoblastic lymphoma	2014
Nucala	Asthma	2015
Cotellic	Melanoma	2015
Aristada	Schizophrenia	2015
Lonsurf	Colorectal cancer	2015
Repatha	Hypercholesterolemia	2015
Daklinza	Hepatitis C	2015
Rexulti	Schizophrenia	2015
Orkambi	Cystic fibrosis	2015
Ibrance	Breast cancer	2015
Alecensa	Non-small cell lung cancer	2015
Tagrissco	Non-small cell lung cancer	2015
Breyanzi	Lymphoma and multiple myeloma	2016
Abecma	Lymphoma and multiple myeloma	2016
Tecartus	Acute lymphoblastic leukemia	2021
Cibinqo	Atopic dermatitis	2022
Kimmtrak	Uveal melanoma​	2022
Casgevy	Sickle cell disease	2023
Elevidys	Duchenne muscular dystrophy	2023
Qalsody	Amyotrophic lateral sclerosis (ALS)	2023
Leqembi	Alzheimer's disease	2023

Regulatory Challenges in Personalized Medicine

The FDA oversees personalized medicine products through three medical product review centers: the Center for Drug Evaluation and Research (CDER), the Center for Devices and Radiological Health (CDRH), and the Center for Biologics Evaluation and Research (CBER). Each center enforces regulations based on statutory authorities, many of which have been established over a long period. However, these existing regulations do not fully address the complexities of personalized medicine, where different types of products rely on each other for safety and efficacy. Consequently, there are inconsistencies in the regulation of personalized medicine products [14].

To address these gaps, the FDA is defining processes and policies within each center's framework to ensure clarity in oversight activities. These efforts aim to streamline premarket reviews of diagnostic products, enhancing consistency and efficiency in decision-making. Assays and other in vivo procedures like electroencephalography, electrocardiography, and diagnostic imaging equipment are examples of diagnostic instruments.

The team focusing on personalized medicine is developing strategies for creating diagnostic tools alongside ethical considerations in clinical pharmacogenomics research, suggesting ways for the FDA to streamline internal collaboration for more effective joint reviews. Additionally, they are assisting in determining the appropriate regulatory balance across centers overseeing personalized medicine products.

Ethical challenges

There are also issues with privacy, ethics, and security that need to be addressed [15-16]. Some of these are highlighted in Table 3 [1].

**Table 3:** Some ethical and policy issues relevant to personalized medicine

Ethical and policy issues	Key considerations and actions
Fairness in access to genomic technologies	Ensuring equitable distribution of genomic tests and therapies based on need rather than financial means.
Intellectual property	Addressing ownership and patent issues to balance innovation with affordable access to genetic technologies.
Regulatory oversight	Establish clear guidelines for safe and ethical use of genetic testing and therapies.
Reimbursement and Insurance	Develop policies to cover costs, ensuring accessibility for all patients.
Education (patient and provider)	Provide comprehensive education on the benefits, risks, and implications of genetic information.
Healthcare system infrastructure	Invest in infrastructure to integrate genetic testing into routine care effectively.
Research and development (R&D) incentives	Create incentives for research on genetic therapies while ensuring affordability.
Management of limited alternatives	Establish ethical guidelines for decision-making when conventional treatments are inadequate.
Consequences of not performing available tests	Address health risks and missed treatment opportunities without genetic testing.
Privacy and confidentiality of genomic information	Implement robust policies to safeguard genetic data from unauthorized access.
Fair use of genomic information by insurers, employers, courts, schools, adoption agencies, and the military	Ensure legal protections against discrimination based on genetic data.
Psychological impact and stigma from misunderstanding pharmacogenomics information	Provide support to mitigate stigma and ensure informed decision-making.
Uncertainties and misunderstandings about gene tests	Educate stakeholders on the limitations and benefits of genetic testing.

Early examples of personalized medicine

Numerous interventions have been developed to suit individual patient characteristics, primarily relying on molecular information. Before discussing specific examples, it is significant to remember that early identification and prevention, in addition to illness treatment, are possible with personalized medicine. This section will address some cases of personalized treatments for diseases. The subsequent section will cover early detection and prevention, as advancements in personalized disease prevention and detection are relatively recent [17].

Many early examples of personalized medications were linked to factors influencing drug metabolism. This was partly due to the established understanding of drug-metabolizing enzymes and their impact on the body's response to medications. A comprehensive resource on the aspects of drug response, including genetic variations affecting drug efficacy and side effects, particularly those related to drug-metabolizing enzymes, can be found in Weber's book.

A notable example is warfarin, a commonly used anticoagulant that, if not administered correctly, can result in serious adverse reactions. Warfarin interacts with genes such as VKORC1 and is partially metabolized by CYP2C9. Naturally occurring variations in these genes contribute to individual differences in warfarin's effectiveness and metabolism. To address this variability, the FDA recommends tailoring warfarin dosages according to a person's genetic composition, specifically their VKORC1 and CYP2C9 gene variants [18].

Another notable example frequently cited in discussions of personalized medicine is the medication imatinib, used in the treatment of CML. Imatinib functions by targeting an enzyme called tyrosine kinase, which becomes overactive due to the fusion of two genetic regions: the Abelson proto-oncogene (abl) and the breakpoint cluster region (bcr). This fusion event, known as the BCR-ABL fusion or the 'Philadelphia chromosome,' is commonly found in tumors contributing to CML development. However, not every person with a CML diagnosis has this fusion mutation present in their tumors. As a result, imatinib is usually limited to CML patients who have a particular fusion event [19].

Future perspectives

The future of personalized medicine is poised for significant advancements, driven by continuous progress in genomics, biotechnology, and data analytics. These developments promise to enhance the precision of healthcare, enabling more individualized and effective treatment strategies.

Advancements in Genomic Technologies

As genomic sequencing becomes increasingly affordable and efficient, the integration of comprehensive genetic profiles into clinical practice will become routine. Whole-genome sequencing and other advanced techniques will deepen our understanding of the genetic underpinnings of diseases, facilitating the development of highly targeted therapies. This will not only enhance treatment efficacy but also enable the identification of genetic predispositions to various conditions, allowing for earlier interventions and personalized preventive strategies.

Integration of Big Data and AI

The convergence of big data analytics and AI with personalized medicine holds immense promise. Large-scale genomic, clinical, and lifestyle data can be analyzed by AI algorithms to find trends and forecast patient reactions to treatments with previously unheard-of accuracy. Machine learning models will assist in identifying novel biomarkers and therapeutic targets, leading to the development of new precision medicines. Furthermore, AI-powered decision support systems will help doctors make better, faster treatment decisions, improving patient outcomes.

Expansion of Personalized Medicine Beyond Oncology

While oncology has been at the forefront of personalized medicine, this approach is expected to expand significantly into other therapeutic areas. Cardiovascular diseases, neurological disorders, and infectious diseases are among the fields where personalized strategies will likely see substantial growth. For instance, pharmacogenomics will play a crucial role in optimizing treatments for cardiovascular conditions by tailoring drug regimens based on individual genetic profiles, thereby reducing adverse effects and improving therapeutic efficacy.

Enhanced Regulatory Frameworks and Ethical Considerations

As personalized medicine evolves, regulatory frameworks must adapt to address the complexities of multi-component therapies and diagnostic tools. Regulatory bodies like the FDA will need to develop more integrated and flexible guidelines that ensure the safety and efficacy of personalized treatments while fostering innovation. Additionally, ethical considerations such as data privacy, equitable access to personalized therapies, and informed consent will become increasingly important. Policymakers and healthcare providers must work together to establish robust safeguards that protect patient rights and promote fairness in the delivery of personalized healthcare.

Education and Training for Healthcare Providers

The successful implementation of personalized medicine will require ongoing healthcare providers' education and training. Medical curricula must be updated to include genomic literacy and the principles of precision medicine. Continuous professional development programs will ensure that clinicians remain abreast of the latest advancements and are equipped to apply personalized approaches effectively in their practice.

Collaborative Research and Global Initiatives

The future of personalized medicine will be shaped by collaborative research efforts and global initiatives. International consortiums and partnerships will enable the sharing of genomic data and clinical insights, accelerating the discovery of new biomarkers and therapeutic targets. Collaborative projects will also promote the development of standardized protocols and best practices, ensuring consistency and quality in personalized medicine applications worldwide.

## Conclusions

A revolutionary development in healthcare is personalized medicine, providing the chance to completely transform medical diagnosis, treatment, and prevention through highly customized methods. Evidence includes the increasing number of drug approvals specifically for personalized treatments, over one-third in 2023, and the proven success of targeted therapies, such as those used in cancer treatments, which have significantly improved patient outcomes. By integrating genomic information with clinical and familial histories, personalized medicine enhances and reduces adverse reactions, and treatment efficacy, and improves patient satisfaction. The ongoing advancements in genomics, biotechnology, and data analytics will continue to drive the evolution of this field, enabling the creation of specialized treatments and diagnostic equipment based on the individual traits of every patient.

The applications of personalized medicine in conditions such as lung cancer, renal carcinoma, and rheumatoid arthritis demonstrate its capacity to significantly enhance patient results. The increasing number of FDA approvals for personalized treatments underscores the growing recognition of this approach's value. However, the successful implementation of personalized medicine will require addressing regulatory challenges, ethical considerations, and the need for ongoing instruction and preparation for medical professionals.

As we look to the future, it is clear that personalized medicine will play a vital part in shaping the next generation of healthcare. By continuing to advance our understanding of genomics and leveraging cutting-edge technologies, we can design a healthcare system that is more accurate, efficient, and patient-centered and that caters to the requirements of both individuals and populations.
